# Chemical Profile of *Cyperus laevigatus* and Its Protective Effects against Thioacetamide-Induced Hepatorenal Toxicity in Rats

**DOI:** 10.3390/molecules27196470

**Published:** 2022-10-01

**Authors:** Iriny M. Ayoub, Marawan A. El-Baset, Mai M. Elghonemy, Samir A. E. Bashandy, Fatma A. A. Ibrahim, Omar A. H. Ahmed-Farid, Abd El-Nasser G. El Gendy, Sherif M. Afifi, Tuba Esatbeyoglu, Abdel Razik H. Farrag, Mohamed A. Farag, Abdelsamed I. Elshamy

**Affiliations:** 1 Department of Pharmacognosy, Faculty of Pharmacy, Ain Shams University, Cairo 11566, Egypt; 2 Pharmacology Department, Medical Research and Clinical Studies Institute, National Research Centre, 33 El-Bohouth St., Dokki, Cairo 12622, Egypt; 3 Department of Natural Compounds Chemistry, National Research Center, 33 El Bohouth St., Dokki, Giza 12622, Egypt; 4 Biochemistry Department, Biotechnology Research Institute, National Research Centre, 33 EL Bohouth St., Dokki, Cairo 12622, Egypt; 5 Physiology Department, National Organization for Drug Control and Research, Cairo 12311, Egypt; 6 Medicinal and Aromatic Plants Research Department, National Research Centre, 33 El Bohouth St., Dokki, Giza 12622, Egypt; 7 Pharmacognosy Department, Faculty of Pharmacy, University of Sadat City, Sadat City 32897, Egypt; 8 Institute of Food Science and Human Nutrition, Department of Food Development and Food Quality, Gottfried Wilhelm Leibniz University Hannover, Am Kleinen Felde 30, 30167 Hannover, Germany; 9 Pathology Department, National Research Centre, 33 El Bohouth St., Dokki, Giza 12622, Egypt; 10 Pharmacognosy Department, Faculty of Pharmacy, Cairo University, Kasr el Aini St., Cairo 11562, Egypt

**Keywords:** smooth flatsedge, flavonoids, aurones, oxidative stress, hepatorenal injuries, inflammation markers, histopathology

## Abstract

*Cyperus* species represent a group of cosmopolitan plants used in folk medicine to treat several diseases. In the current study, the phytochemical profile of *Cyperus laevigatus* ethanolic extract (CLEE) was assessed using UPLC-QTOF–MS/MS. The protective effect of CLEE at 50 and 100 mg /kg body weight (b.w.) was evaluated on hepatorenal injuries induced by thioacetamide (100 mg/kg) via investigation of the extract’s effects on oxidative stress, inflammatory markers and histopathological changes in the liver and kidney. UPLC-QTOF–MS/MS analysis of CLEE resulted in the identification of 94 compounds, including organic and phenolic acids, flavones, aurones, and fatty acids. CLEE improved the antioxidant status in the liver and kidney, as manifested by enhancement of reduced glutathione (GSH) and coenzyme Q10 (CoQ10), in addition to the reduction in malondialdehyde (MDA), nitric oxide (NO), and 8-hydroxy-2′-deoxyguanosine (8OHdG). Moreover, CLEE positively affected oxidative stress parameters in plasma and thwarted the depletion of hepatorenal ATP content by thioacetamide (TAA). Furthermore, treatment of rats with CLEE alleviated the significant increase in plasma liver enzymes, kidney function parameters, and inflammatory markers. The protective effect of CLEE was confirmed by a histopathological study of the liver and kidney. Our results proposed that CLEE may reduce TAA-hepatorenal toxicity via its antioxidant and anti-inflammatory properties suppressing oxidative stress.

## 1. Introduction

Medicinal plants represent the most enriched resources for natural remedies worldwide [1,2,3]. Recently, medicinal plants and their byproducts have proved to be the main source of safe medicinal drugs because of their bioactive secondary metabolites [2,4]. Moreover, plants provide phytochemicals to manage and protect against a multitude of diseases, owing to minimal side effects, and/or low toxicity and costs, along with significant biological potentiality and availability [4]. All documented data reported a strong relationship between the biological activities, including anti-inflammatory, antioxidant, and hepatoprotective potentialities of medicinal herbs, and their bioactive metabolites [2]. Plant phytochemicals, including phenolic acids, organic acids, flavonoids, aurones, and others, are the main contributors to the hepatoprotective mechanism [4].

Plants belonging to the *Cyperus* genus (family Cyperaceae) are widely distributed around the world with several traditional uses [5,6]. Various byproducts from *Cyperus* plants displayed several potent biological and pharmaceutical potentialities, including anti-inflammatory [7], hepatoprotective [8], anti-ulcer [5,6], antimalarial [9], and antidiabetic [10] activities. Many secondary metabolites were identified in *Cyperus* plants, including phenolic acids, flavonoids, aurones, quinones [5,6,11,12], steroids, terpenes [13], and alkaloids [14], along with volatile oils [15,16]. The previous work on *C. laevigatus* aerial parts resulted in the characterization of some phenolic acids and flavonoids [7] alongside volatile components [16]. Essential oil, extracts, and metabolites derived from this plant were reported to possess significant antioxidant, anti-inflammation, antimicrobial, and anti-diabetic effects [7,16].

Thioacetamide (TAA), an organo-sulfur white crystal, has been reported to cause carcinogenic damage to the liver and thus used to induce rats’ hepatic injury [17]. The documented toxicological reports deduced the neurotoxic effect of TAA in animals and humans [18]. TAA may cause the generation of reactive oxygen species (ROS), destroying free radical scavenging mechanisms and some cellular ingredients such as DNA, lipids, and proteins, thus deteriorating cellular structures and functions [19].

The current study provides mechanistic insights on potential protective effects of CLEE against hepatorenal damage induced by thioacetamide. Comprehensive metabolites profiling of *C. laevigatus* ethanolic extract (CLEE) was described herein for the first time using UPLC-ESI–QTOF-MS/MS. Additionally, the protective effect of CLEE against thioacetamide-induced hepatorenal toxicity in rat models was assessed based upon biochemical and histochemical results.

## 2. Results

### 2.1. Identification of Bioactive Compounds

**Figure 1:**
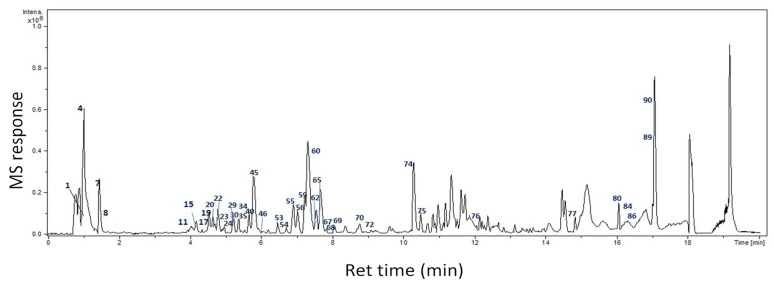
UPLC-QTOF–MS base peak chromatogram of CLEE in negative ion mode. Peaks are numbered following that listed in Table 1.

**Figure 2:**
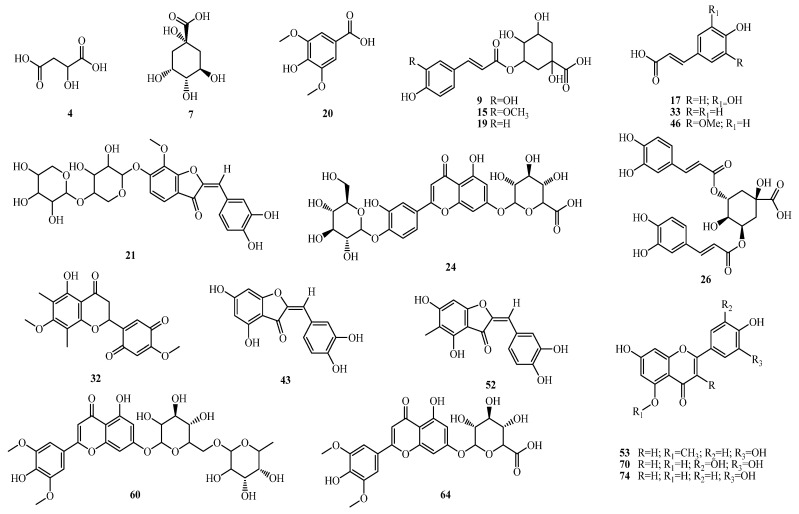
Representative structures of major metabolites identified in CLEE and peak names follow the numbers stated in Table 1.

UPLC-ESI-QTOF–MS/MS analysis of the ethanolic extract was performed to characterize the bioactive compounds of CLEE. A total of 94 compounds belonging to various classes were annotated, including organic and phenolic acids, flavones, aurones, and fatty acids. The base peak chromatogram is depicted in Figure 1 in negative ion mode. Compounds were identified based on molecular formula, MS/MS fragmentation pattern, the reported data in the literature, and the phytochemical dictionary of natural products, as listed in Table 1 and Figure 2, representing the first comprehensive phytochemical profiling of CLEE.

#### 2.1.1. Organic Acids

Organic acids were abundant in CLEE, represented by gluconic acid (**1**), tetrahydroxypentanoic acid (**2**), malic acid (**4**), and its isomer (**5**), in addition to fumaric acid (**6**), quinic acid (**7**), and citric/isocitric acid (**8**), by comparison with published data [5,20]. Malic acid amounted to the major organic acid.

**Table 1:** Metabolites profiling of CLEE via UPLC-QTOF–MS/MS analysis in negative ion mode.

Peak No	Name	MF	Rt	[M−H]^−^ m/z	Diff (ppm)	Ms^2^	Class	Ref.
1	Gluconic acid	C_6_H_12_O_7_	0.968	195.0500	5.1	161, 129, 89, 75	Organic acid	
2	Tetrahydroxypentanoic acid	C_5_H_10_O_6_	0.980	165.0405	5.3	147, 99, 87, 75	Organic acid	[5]
3	Hexose	C_6_H_12_O_6_	0.988	179.0562	−0.5	161, 131, 113, 85	Sugar	
4	Malic acid	C_4_H_6_O_5_	1.033	133.0145	−1.8	115, 89	Organic acid	
5	Malic acid isomer	C_4_H_6_O_5_	1.308	133.0141	0.8	115	Organic acid	
6	Fumaric acid	C_4_H_4_O_4_	1.346	115.0037	0.3	69	Organic acid	
7	Quinic acid	C_7_H_12_O_6_	1.438	191.0566	−2.5	111, 87	Organic acid	
8	Citric acid/Isocitric acid	C_6_H_8_O_7_	1.514	191.0208	−5.7	155, 129, 111	Organic acid	
9	*O*-Caffeoylquinic acid	C_16_H_18_O_9_	3.479	353.0858	5.7	191	Phenolic acid	
10	Unknown	C_14_H_24_O_10_	4.300	351.1297	3.5	113, 101, 89	Unknown glycoside	
11	*O*-Caffeoylquinic acid isomer	C_16_H_18_O_9_	4.028	353.0877	−0.3	191	Phenolic acid	
12	*O*-Coumaroylhexose	C_15_H_18_O_8_	4.120	325.0932	−1.0	191, 179	Phenolic acid	
13	Asperuloside	C_18_H_22_O_11_	4.202	413.1659	−1.4	353, 345, 267, 249	Iridoid	
14	Hydroxybenzoic acid	C_7_H_6_O_3_	4.134	137.0244	1.2	93	Phenolic acid	
15	Feruloyl quinic acid	C_17_H_20_O_9_	4.148	367.1035	2.0	193	Phenolic acid	
16	Feruloyl-*O*-hexoside	C_16_H_20_O_9_	4.337	355.1035	−0.2	163	Phenolic acid	
17	Caffeic acid	C_9_H_8_O_4_	4.422	179.0350	−9.2	135	Phenolic acid	
18	*O*-Syringoyl quinic acid	C_16_H_20_O_10_	4.464	371.0988	−1.2	323, 305, 121	Phenolic acid	
19	Coumaroyl quinic acid	C_16_H_18_O_8_	4.543	337.0935	−1.8	191, 173	Phenolic acid	
20	Syringic acid	C_9_H_10_O_5_	4.598	197.0460	−2.5	182, 167	Phenolic acid	
21	Leptosidin-*O*-dipentoside (Tetrahydroxy-methoxy-aurone-*O*-di-pentoside)	C_26_H_28_O_14_	4.557	563.1411	−0.8	nd	Aurone	[21]
22	Feruloylquinic acid isomer	C_17_H_20_O_9_	4.763	367.1035	−2.3	193, 173	Phenolic acid	[5]
23	Luteolin di-*O*-hexoside	C_27_H_30_O_16_	4.852	609.1459	0.4	447	Flavone	[22]
24	Luteolin- *O*-hexoside-*O*-glucuronide	C_27_H_28_O_17_	4.900	623.1236	2.9	461	Flavone	
25	*O*-Syringoylquinic acid *O*-(Hydroxydimethoxy- benzoyl)-quinic acid	C_16_H_20_O_10_	4.952	371.0984	2.5	353, 327, 249, 231, 121	Phenolic acid	[5]
26	Dicaffeoylquinic acid	C_25_H_24_O_12_	4.918	515.1213	−3.4	Nd	Phenolic acid	[5]
27	*O*-(Hydroxydimethoxybenzoyl)-quinic acid isomer	C_16_H_20_O_10_	4.969	371.0978	1.7	249	Phenolic acid	
28	*O*-Coumaroylglycerol	C_12_H_14_O_5_	5.097	237.0768	2.1	145	Phenolic acid	
29	*O*-Caffeoyl-*O*-syringoylquinic acid	C_25_H_26_O_13_	5.193	533.1301	−1.6	469, 443, 255	Phenolic acid	
30	Tetrahydroxy-dimethoxyflavone- di-*O*-hexoside	C_29_H_34_O_18_	5.364	669.1657	2.3	623, 619, 507, 427	Flavone	
31	Tetrahydroxy-methoxyflavone- dihexoside	C_28_H_32_O_17_	5.282	639.1567	0.3	593	Flavone	
32	5-Hydroxy-4′,7-dimethoxy-6,8-dimethyl-2′,5′-flavanonequinone (Scaberin)	C_19_H_18_O_7_	5.357	357.0980	−2.5	163, 119	Flavanone quinone	[6]
33	Hydroxycinnamic acid	C_9_H_8_O_3_	5.388	163.0403	−1.2	119	Phenolic acid	[23]
34	Dihydroxy-dimethoxy-methylaurone	C_18_H_16_O_6_	5.411	327.0874	−1.6	283, 163	Aurone	[24]
35	Tetrahydroxy-dimethoxyflavone- di-*O*-hexoside	C_29_H_34_O_18_	5.415	669.1674	−0.3	619, 507, 427	Flavone	
36	Hydroxycinnamic acid isomer	C_9_H_8_O_3_	5.532	163.0405	−2.4	119	Phenolic acid	[23]
37	Luteolin-*O*-deoxyhexoside -*O*-glucuronide	C_27_H_28_O_16_	5.473	607.1305	−0.1	493, 436, 284	Flavone	
38	Hydroxyoctanoic acid-*O*-hexoside	C_14_H_26_O_8_	5.432	321.1555	5.2	158, 114	Fatty acid	
39	Hydroxycinnamoyl-*O*-malic acid	C_13_H_12_O_7_	5.515	279.0510	−2.2	163	Phenolic acid	
40	Tetrahydroxyflavone-*O*-pentosyl hexoside (Luteolin-*O*-pentosyl hexoside)	C_36_H_36_O_7_	5.649	579.2388	−1.9	493, 285, 284	Flavone	
41	Tetrahydroxyaurone-*O*-glucuronide (Aureusidin 6-*O*-glucuronide)	C_21_H_18_O_12_	5.748	461.0725	−2.9	285, 283	Aurone	[24]
42	Tetrahydroxyaurone-*O*-glucuronide isomer	C_21_H_18_O_12_	5.763	461.0736	−2.2	285	Aurone	
43	Aureusidin (Tetrahydroxyaurone)	C_15_H_10_O_6_	5.820	285.0405	−2.6	Nd	Aurone	[22]
44	Tetrahydroxymethoxyflavone-*O*-glucuronide	C_22_H_20_O_13_	5.903	491.0831	−2.0	315	Flavone	
45	Pentahydroxymethoxyflavone-*O*-glucuronide	C_22_H_20_O_14_	5.910	507.0780	−2.7	462, 331	Flavone	
46	Ferulic acid (Hydroxymethoxycinnamic acid)	C_10_H_10_O_4_	5.937	193.0514	−3.8	178, 134	Phenolic acid	[5]
47	Hydroxydimethoxycinna-mic acid isomer	C_11_H_12_O_5_	5.947	223.0612	−2.2	nd	Phenolic acid	
48	Tetrahydroxymethoxyflavone-*O*-glucuronide isomer	C_22_H_20_O_13_	5.995	491.0831	−1.5	315, 175	Flavone	
49	Hydroxymethoxycinnamic acid isomer	C_10_H_10_O_4_	5.999	193.0506	−2.5	178, 134	Phenolic acid	[5]
50	Luteolin-*O*-glucuronide (Tetrahydroxy-flavone- *O*-glucuronide)	C_21_H_18_O_12_	6.016	461.0742	−3.5	325, 285	Flavone	[22]
51	Dicaffeoylquinic acid isomer	C_25_H_24_O_12_	6.164	515.1195	−1.1	353	Phenolic acid	[5]
52	Tetrahydroxy-methylaurone	C_16_H_12_O_6_	6.449	299.0561	−4.8	284, 267	Aurone	[22,23,24]
53	Luteolin-5-methyl ether (Trihydroxymethoxyflavone)	C_16_H_12_O_6_	6.507	299.0561	−5.3	283, 267, 238, 147	Flavone	[24]
54	Luteolin methyl ether glucuronide (Trihydroxymethoxyflavone glucuronide)	C_22_H_20_O_12_	6.579	475.0889	−1.5	299	Flavone	[24]
55	Tricin (Trihydroxy-dimethoxyflavone)	C_17_H_14_O_7_	6.713	329.0681	−4.3	261	Flavone	[22]
56	Tricin (7-*O*-deoxhexosyl-*O*-glucuronide)	C_29_H_32_O_17_	7.035	651.1575	−1.3	329	Flavone	
57	Luteolin-*O*-deoxyhexoside	C_21_H_20_O_10_	7.104	431.1003	−4.4	285	Flavone	
58	Trihydroxyflavone-*O*-glucuronide (Apigenin glucuronide)	C_21_H_18_O_11_	7.189	445.0786	−2.2	269, 175		[14]
59	Trihydroxyflavone-*O*-glucuronide isomer	C_21_H_18_O_11_	7.257	445.0776	−5.0	269, 175	Flavone	
60	Tricin-7-*O*-deoxyhexosyl-*O*-hexoside	C_29_H_34_O_16_	7.346	637.1774	−3.1	593, 447, 329	Flavone	
61	Tricin-7-*O*-hexosyl sulfate	C_23_H_24_O_15_S	7.397	571.0768	−0.8	329, 255, 241, 175	Flavone	[25]
62	Tricin-7-*O*-hexosyl sulfate isomer	C_23_H_24_O_15_S	7.455	571.0767	−1.8	329, 271	Flavone	[24]
63	Tetrahydroxy-methoxyflavone-*O*-sulfate (Methoxyluteolin-*O*-sulfate)	C_16_H_12_O_10_S	7.575	395.0078	−4.9	315, 225, 209, 167	Flavone	
64	Tricin-7-*O*-glucuronide	C_23_H_22_O_13_	7.616	505.0988	1.2	329	Flavone	[23]
65	Luteolin methyl ether-*O*-glucuronide isomer	C_22_H_20_O_12_	7.647	475.0905	−1.2	299, 177, 175	Flavone	
66	Tetrahydroxyaurone-*O*-hexoside	C_21_H_20_O_11_	7.831	447.0954	−4.7	411, 285, 233	Aurone	[14]
67	Tetrahydroxyflavone-*O*-hexoside (Luteolin-*O*-hexoside)	C_21_H_20_O_11_	7.875	447.0933	1.1	285, 255	Flavone	[26]
68	Dihydroxymethoxyaurone	C_16_H_12_O_5_	8.053	283.0631	−6.6	268, 240	Aurone	
69	Dihydroxymethoxyflavone	C_16_H_12_O_5_	8.104	283.0626	−4.9	269, 147	Flavone	
70	Pentahydroxyflavone (Tricetin)	C_15_H_10_O_7_	8.128	301.0360	−2.2	nd	Flavone	[23,27]
71	Trihydroxy-methoxy-methylaurone	C_17_H_14_O_6_	8.803	313.0718	−0.2	298, 267	Aurone	
72	Hydroxy-dodecenedioic acid	C_12_H_20_O_5_	9.201	243.1250	−4.8	225, 199	Fatty acid	
73	Dihydroxy-decenoic acid	C_10_H_18_O_4_	10.200	201.1120	6.2	183, 139	Fatty acid	
74	Tetrahydroxyflavone (Luteolin)	C_15_H_10_O_6_	10.251	285.0405	−0.3	nd	Flavone	
75	Trihydroxy-methoxy-flavonol (Isorhamnetin)	C_16_H_12_O_7_	10.389	315.0518	−2.4	300, 271, 175	Flavonol	[22]
76	Hydroxyoctadecenedioic acid	C_18_H_32_O_5_	11.953	327.2196	−6.0	309, 239	Fatty acid	
77	Hydroxyoctadecadienoic acid	C_18_H_32_O_3_	14.767	295.2292	−4.4	277, 237	Fatty acid	
78	Hydroxytetradecanoic acid	C_14_H_28_O_3_	15.827	243.1963	1.1	nd	Fatty acid	
79	Hydroxydocosanoic acid	C_22_H_44_O_3_	15.878	355.3211	1.8	nd	Fatty acid	
80	Hydroxytetracosenoic acid	C_24_H_46_O_3_	16.003	381.3364	2.8	nd	Fatty acid	
81	Hydroxypentadecanoic acid	C_15_H_30_O_3_	16.409	257.2124	−0.6	nd	Fatty acid	
82	Hydroxyhexadecenoic acid	C_16_H_30_O_3_	16.413	269.2131	−3.2	225	Fatty acid	
83	Hydroxyeicosanoic acid	C_20_H_40_O_3_	16.478	327.2894	3.2	nd	Fatty acid	
84	Eicosanoic acid (Arachidic acid)	C_20_H_40_O_2_	16.491	311.2957	−0.5	nd	Fatty acid	
85	Hexacosanoic acid	C_26_H_52_O_2_	16.642	395.3886	2.0	nd	Fatty acid	
86	Hydroxytetracosanoic acid	C_24_H_48_O_3_	16.666	383.3531	1.3	nd	Fatty acid	
87	Octadecanoic acid	C_18_H_36_O_2_	16.734	283.2626	5.9	nd	Fatty acid	
88	Octadecatrienoic acid	C_18_H_30_O_2_	16.799	277.2186	−4.6	nd	Fatty acid	
89	Trihydroxy octadecenoic acid	C_18_H_34_O_5_	16.876	329.2326	2.3	nd	Fatty acid	
90	Hydroxypalmitic acid	C_16_H_32_O_3_	17.049	271.2270	3.3	225	Fatty acid	
91	Hexadecenoic acid	C_16_H_30_O_2_	17.244	253.2173	−3.8	nd	Fatty acid	
92	Docosanedioic acid	C_22_H_42_O_4_	17.368	369.3004	1.4	nd	Fatty acid	
93	Pentacosanedioic acid	C_25_H_48_O_4_	17.479	411.3470	2.4	nd	Fatty acid	
94	Eicosanedioic acid	C_20_H_38_O_4_	17.660	341.2685	3.4	nd	Fatty acid	

#### 2.1.2. Phenolic Acids

Several phenolic acids were annotated, including hydroxybenzoic acid (**14**), displaying a characteristic 44 amu (CO_2_) loss. Syringic acid (**20**) exhibited a molecular ion [M–H]^−^ at *m/z* 197.0460 and yielded a product ion at *m/z* 167, indicating the loss of two methyl groups. In addition, several hydroxycinnamic acids were annotated in CLEE, including caffeic and ferulic acid conjugates. Caffeic acid (**17**) [M−H]^−^ at *m/z* 179.0350 was characterized by a loss of a CO_2_ group forming a product ion at *m/z* 135. Ferulic acid (**46**) and its isomer (**49**) showed deprotonated molecular ions at *m/z* 193.0514 and 193.0506, respectively, followed by a subsequent loss of CH_3_-group yielding a product ion [M–H−15]^−^ at *m/z* 178. Aside from free cinnamates, acylated forms were detected. For example, caffeoyl quinic acid (**9**) and its isomer (**11**) showed a pseudomolecular ion at *m/z* 353.0858 and 353.0877, respectively, and displayed a fragment ion of quinic acid moiety (*m/z* 191) [28]. Peaks (**26**) and (**51**) were annotated as dicaffeoylquinic acid, exhibiting quasi-molecular ions at *m/z* 515.1213 and 515.1195, respectively, and a product ion at *m/z* 353 corresponding to the loss of a caffeoyl moiety.

#### 2.1.3. Flavonoids

Various flavonoids were detected, including flavones, flavonols, and aurones, with flavones amounting to the major subclass. Luteolin, luteolin 5-methyl ether, and tricin-*O*-glycosides were the major flavones identified in CLEE. This concurs with previous reports on *Cyperus* species [24]. Likewise, members of Cyperaceae exhibited the same pattern, especially in the occurrence of the rare marker compound, luteolin 5-methyl ether. Additionally, tricin, and luteolin were found in the glycosidic form in conjugation with uronic acids exemplified in 7-glucosides and/or 7-glucuronides [22,25]. Luteolin was mainly found as luteolin-*O*-glucuronide (**50**), exhibiting a deprotonated ion at *m*/*z* 461.0742 and a predominant product ion at *m*/*z* 285 [M–H–176]^−^ indicating a glucuronyl unit loss. Other luteolin glycosides were identified in peaks (**57**) at *m/z* 431.1003, (**67**) *m/z* 447.0933, (**40**) *m/z* 579.2388, (**37)** *m/z* 607.1305, (**23**) *m/z* 609.1459, and (**24**) *m/z* 623.1236. In MS–MS spectrum, the most abundant fragment ion at *m*/*z* 285 was observed corresponding to [M–H–146]^−^, [M–H–162]^−^, [M–H–132–162]^−^, [M–H–146–176]^−^, [M–H–162–162]^−^, and [M–H–162–176]^−^, respectively. These glycosides were identified as luteolin-*O*-deoxyhexoside (**57**), luteolin-*O*-hexoside (**67**), luteolin-*O*-pentosyl hexoside (**40**), luteolin-*O*-deoxyhexoside-*O*-glucuronide (**37),** luteolin di-*O*-hexoside (**23**), and luteolin-*O*-hexoside-*O*-glucuronide (**24**). Likewise, luteolin 5-methyl ether (**53**) was detected at *m/z* 299.0561. Additionally, luteolin 5-methyl ether-*O*-glucuronide (**54**, **65**) was characterized by [M–H]^−^ at *m*/*z* 475.0889 and 475.0905, respectively, exhibiting a product ion at *m*/*z* 299 [M–H–176]^−^ indicating a glucuronyl unit loss.

Another flavone, aglycone, tricin (**55**) [M–H]^−^ at *m*/*z* 329.0681, was similarly detected. Other glucuronide/glycosidic conjugates of tricin were detected in peaks (**56** and **64**). In detail, compound (**64**) exhibited [M–H]^−^ at *m/z* 505.0988 and a product ion at *m/z* 329, corresponding to the loss of a glucuronyl unit (176 Da), indicating tricin-7-*O*-glucuronide. Likewise, peak (**56**) [M–H]^−^ at *m/z* 651.1575 showed an additional loss of 146 amu, indicating tricin-7-*O*-deoxhexosyl-*O*-glucuronide. Noteworthy, for tricin glycosides, the glucuronyl moiety was tentatively assigned to the *O*-7 hydroxyl group because the hydroxyl group at C-5 in ring A is not considered a place for sugar substitution [29]. Additionally, the presence of two methyl groups on ring B hydroxyls at C-3′ and C-5′ creates a spatial hindrance for sugar substitution to occur at the C-4′ hydroxyl group [29]. In the same context, compound (**60**) [M − H]^−^ at *m*/*z* 637.1774 showed a fragment ion at *m*/*z* 329, indicating the loss of a deoxyhexose (146 Da) and a hexose (162 Da), and annotated as tricin-7-*O*-deoxyhexosyl-*O*-hexoside_._

Sulfated glycosides are typical components in the Cyperaceae family and could be detected herein mostly based on the loss of 80 amu for sulfate moiety. For example, MS-MS spectra of compounds (**61)** and (**62**) revealed the same predominant product ion at *m*/*z* 329 [M–H–80–162]^−^, thus, indicating the loss of a sulphated hexosyl unit and annotated as tricin-7-*O*-hexosylsulfate in agreement with previous reports [25]. Likewise, compound (**63**) showed a similar pattern displaying a molecular ion at *m/z* 395.0078 [M–H]^−^ C_16_H_12_O_10_S and an abundant product ion at *m/z* 315, indicating the loss of a sulfate moiety (80 Da) and assigned as tetrahydroxymethoxyflavone-*O*-sulfate.

#### 2.1.4. Aurones

Aurones are structural isomers of flavones and are considered minor flavonoids owing to their very limited abundance in nature [22]. Abundant aurones were reported in *Cyperus* species [22]. In the current study, several aurones were identified in peaks **34**, **41**, **42**, **43**, **52**, **66**, **68**, and **71**. Peak assignment was based on the earlier elution of aurones compared to structurally related flavone isomers [30]. Compound (**43**) at *m/z* 285.0405 was tentatively identified as aureusidin, the most widely distributed aurone. Compound (**34**) displayed [M–H]− at *m/z* 327.0874 corresponding to C_18_H_16_O_6_, assigned as dihydroxy-dimethoxy-methylaurone based on product ions at *m*/*z* 283 and 163. Compound (52) [M–H]− at *m/z* 299.0561, C_16_H_12_O_6_ showed daughter fragment ions at *m/z* 284 and 267 and annotated as tetrahydroxy-methylaurone. Likewise, peak (71) displayed [M−H]^−^ at *m/z* 313.0718 (C_17_H_14_O_6_), showing a similar fragmentation behavior to compound (**52**), with an extra 14 amu and annotated as trihydroxy-methoxy-methylaurone [23].

Furthermore, several aurone glycosides were detected in peaks **41** and **42** at *m/z* 461.0742 and *m/z* 461.0736, respectively, with a predicted molecular formula C_21_H_18_O_12_ and exhibited a daughter fragment ion at *m/z* 285 [M–H−176]^−^ annotated as tetrahydroxyaurone-*O*-glucuronide. Peak **21** displayed molecular ion [M−H]^−^ at *m/z* 563.1411 (C_26_H_28_O_14_) and assigned as leptosidin-*O*-dipentoside, previously reported in *C. scariosus* and for the first time reported in CLEE [21].

#### 2.1.5. Fatty Acids

Numerous fatty acids were annotated in CLEE, displaying the intense molecular ion peak [M−H]^−^ typical of fatty acids in the second half of the chromatogram in negative ion mode. A hydroxy unsaturated dicarboxylic fatty acid was detected exhibiting [M−H]^−^ at *m/z* 327.2196 C_18_H_32_O_5_ and displaying losses of a water molecule *m/z* 309 [M−H−H_2_O]^−^, and two CO_2_ molecules *m/z* 239 [M−H−2CO_2_]^−^ indicating a hydroxy dicarboxylic acid annotated as hydroxyoctadecenedioic acid (**76**). Additionally, several monohydroxy saturated fatty acids were observed, including hydroxytetradecanoic acid (**78**) C_14_H_28_O_3_ at *m/z* 243.1963, hydroxydocosanoic acid (**79**) C_22_H_44_O_3_ at *m/z* 355.3211, hydroxypentadecanoic acid (**81**) C_15_H_30_O_3_ at *m/z* 257.2124, hydroxyeicosanoic acid (**83**) C_20_H_40_O_3_ at *m/z* 327.2894, hydroxypalmitic acid (**90**) C_16_H_32_O_3_ at *m/z* 271.2270 and hydroxytetracosanoic acid (**87**) C_24_H_48_O_3_ at *m/z* 383.3531. In addition, monohydroxy unsaturated fatty acids hydroxytetracosenoic acid (**80**) C_24_H_46_O_3_ at *m/z* 381.3364, hydroxyhexadecenoic acid (**82**) C_16_H_30_O_3_ at *m/z* 269.2131 were also assigned. Additionally, a trihydroxy unsaturated fatty acid was identified as trihydroxyoctadecenoic acid (**89**) exhibiting [M−H]^−^ at *m/z* 329.2326 C_18_H_34_O_5_. Noteworthy, fatty acids showing a mass difference of two amu indicate the occurrence of an additional double bond [28]. Octadecatrienoic acid (**88**) C_18_H_30_O_2_ at *m/z* 277.2186 displayed a decrease by six amu from compound (**87**), indicating the occurrence of three double bonds, and hexadecenoic acid (**91**) C_16_H_30_O_2_ at *m/z* 253.2173 could be identified. Additionally, dicarboxylic saturated fatty acids were also detected, including docosanedioic acid (**92**) C_22_H_42_O_4_, pentacosanedioic acid (**93**) C_25_H_48_O_4_ at *m/z* 369.3004 and eicosanedioic acid (**94**) C_20_H_38_O_4_ at *m/z* 341.2685.

### 2.2. Thioacetamide-Induced Hepatorenal Toxicity Results

#### 2.2.1. Body Weight

The administration of TAA led to a marked reduction in the rats’ body weight from the second week until the sixthweek (Table 2) by 19%, 19.5%, 28%, 27%, and 26% compared to the control, respectively. On the contrary, the rats that were given CLEE + TAA exhibited an insignificant gain of body weight until the fifth week. Only CLEE (100 mg/kg) in the sixth week significantly ameliorated the body weight gain by 11% compared to the TAA (*p* ≤ 0.05).

#### 2.2.2. Biochemical Indicators of Hepatic and Renal Function

**Figure 3:**
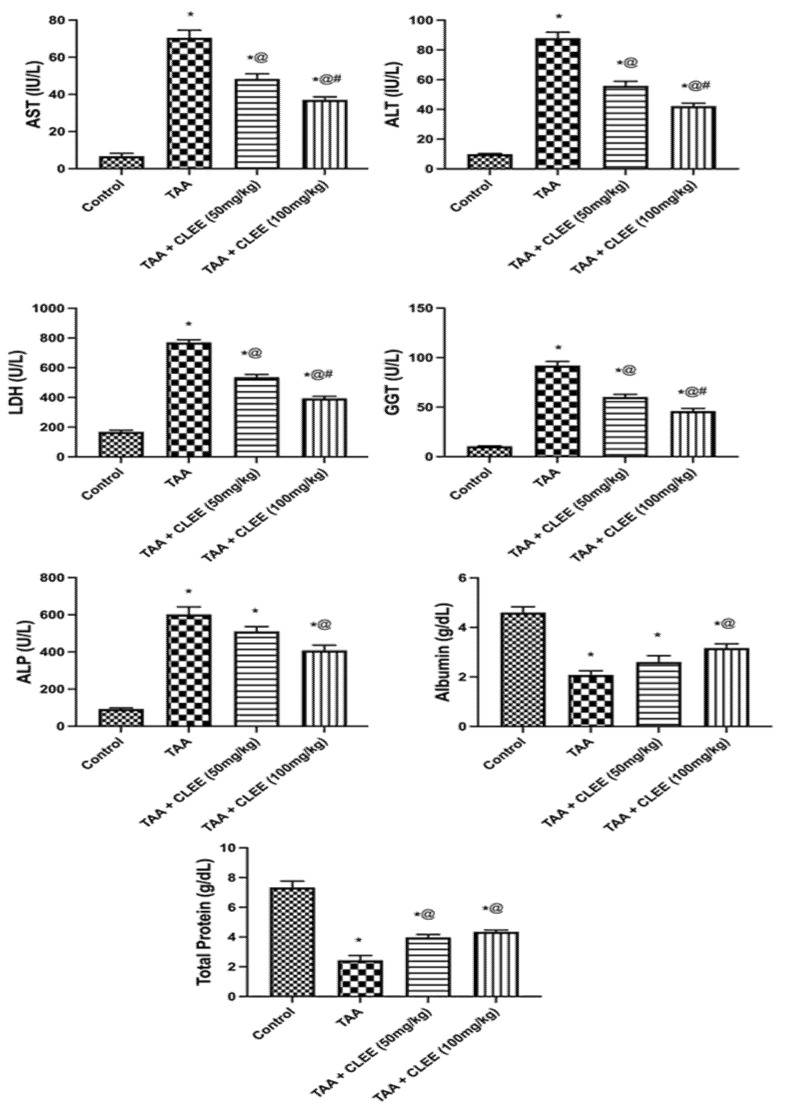
Effect of CLEE on liver function parameters of rats intoxicated by TAA. Each value represents the mean of six animals ± SE. Statistical analysis was performed using one-way ANOVA followed by the Tukey-Kramer multiple comparisons test. (* vs. control group, @ vs. TAA group and ^#^ vs. TAA + 50 mg /kg b.w. CLEE group) at *p* < 0.05.

**Figure 4:**
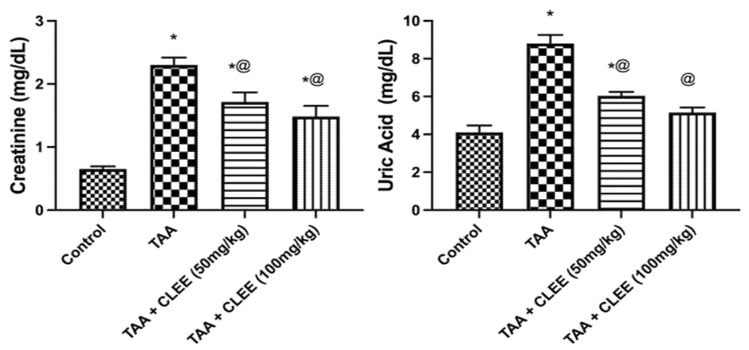
Effect of CLEE on kidney function parameters of rats intoxicated by TAA. Each value represents the mean of six animals ± SE. Statistical analysis was performed using one-way ANOVA followed by the Tukey-Kramer multiple comparisons test. (* vs. control group, @ vs. TAA group) at *p* < 0.05.

The administration of TAA led to a significant elevation (*p* ≤ 0.05) in serum AST, ALT, ALP, GGT, and LDH by 9.0-, 10.5-, 6.4-, 8.7-, and 4.6-fold compared to the control group, respectively. On the contrary CLEE (100 mg/kg) showed a significant reduction in serum AST, ALT, ALP, GGT, and LDH by 52%, 48%, 32%, 49%, and 48%, respectively, compared to the TAA group. Likewise, CLEE (50 mg/kg) displayed a marked reduction in serum AST, ALT, GGT, and LDH by 36%, 31%, 34%, and 30%, respectively, compared to the TAA group. Contrariwise, the total protein and the albumin levels depicted lower levels in the TAA group compared to the control group by 66% and 55%, respectively. Meanwhile, CLEE at 50 and 100 mg/kg restored the serum total protein by 64% and 79%, respectively, compared to the TAA group. Only CLEE (100 mg/kg) replenished the serum albumin by 52% compared to the TAA group (Figure 3). The intoxication by TAA resulted in a significant elevation in serum creatinine and uric acid levels by 3.5- and 2.1-fold, respectively, compared to the control group. Conversely, administration of CLEE at 50 and 100 mg/kg reduced the serum creatinine levels by 25% and 36% and uric acid levels by 31% and 41%, respectively, compared to the TAA group (Figure 4).

#### 2.2.3. Plasma Oxidative Stress and Inflammatory Markers

**Figure 5:**
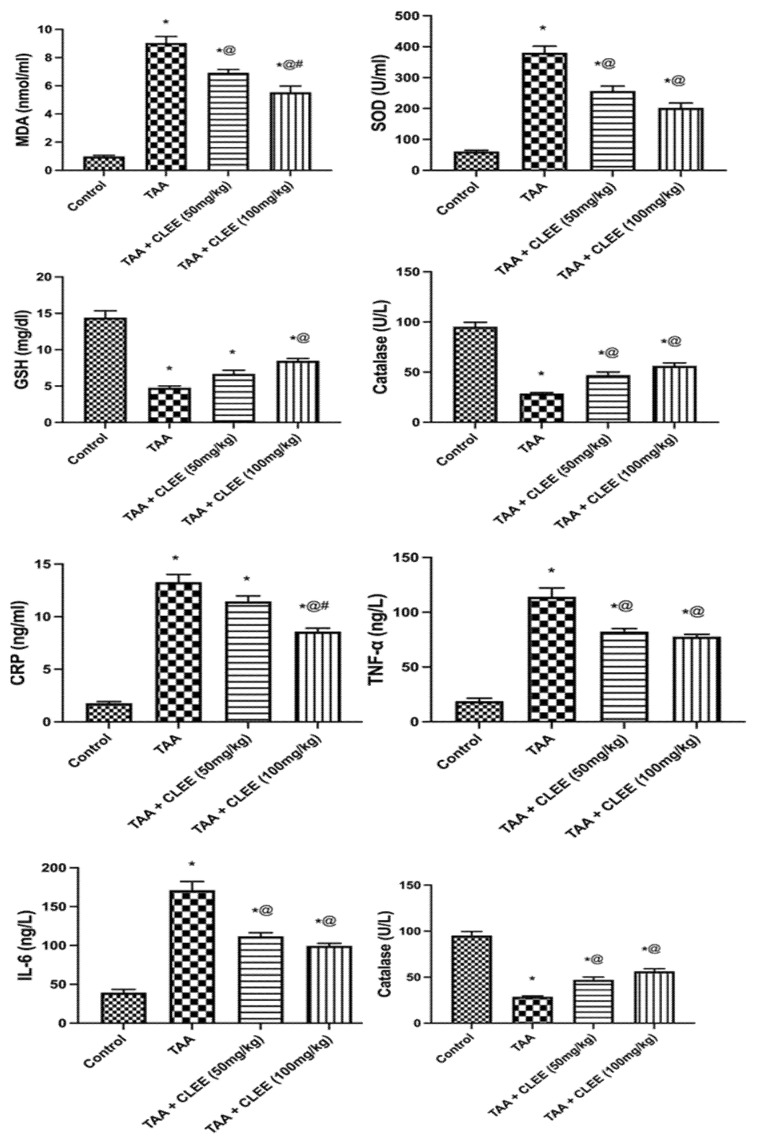
Effect of *C. laevigatus* EtOH extract (CLEE) on plasma oxidative stress and inflammatory parameters of rats intoxicated by TAA. Each value represents the mean of six animals ± SE. Statistical analysis was performed using one-way ANOVA followed by the Tukey-Kramer multiple comparisons test. (* vs. control group, @ vs. TAA group and # vs. TAA + 50 mg /kg b.w. CLEE group) at *p* < 0.05.

The administration of TAA led to a significant increase (*p* ≤ 0.05) in MDA, SOD, CRP, TNF-α, and IL6 levels by 9.2-, 6.2-, 7.4-, 6.0- and 4.4-fold, respectively, compared to the control group. On the other hand, CLEE (50 mg/kg) decreased the previous parameters by 23%, 32%, 14%, 28%, and 35%, respectively, compared to the TAA group. While CLEE (100 mg/kg) reduced the serum level of MDA, SOD, CRP, TNF-α, and IL6 by 38%, 47%, 35%, 32%, and 42%, respectively (Figure 5). The TAA group depicted lower serum levels of GSH and catalase than the control group by 67% and 70%, respectively. On the contrary, CLEE (50 and 100 mg/kg) elevated the GSH by 40% and 80%, respectively, compared to the TAA group. Meanwhile, CLEE (50 and 100 mg/kg) showed increased serum catalase by 1.6- and 2.0-fold, respectively, compared to the TAA group (Figure 5).

#### 2.2.4. Hepatic and Renal Oxidative Stress and Energy Parameters

**Figure 6:**
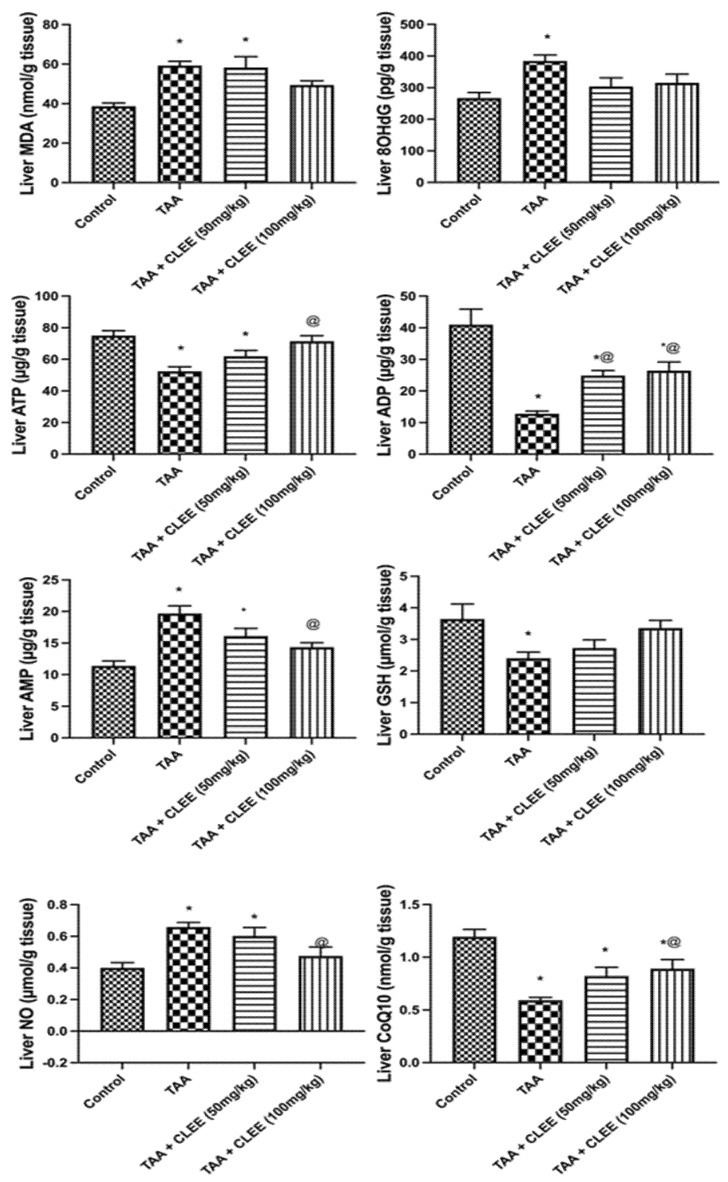
Effect of *C. laevigatus* EtOH extract (CLEE) on hepatic oxidative stress and energy parameters of rats intoxicated by TAA. Each value represents the mean of six animals ± SE. Statistical analysis was performed using one-way ANOVA followed by the Tukey-Kramer multiple comparisons test. (* vs. control group, and @ vs. TAA group) at *p* < 0.05.

Figure 6 indicated that TAA-induced prominent elevation in hepatic content of MDA, 8OHdG, NO, and AMP by 53%, 44%, 73%, and 65%, respectively, compared to the control group. On the other side, only the administration of CLEE (100 mg/kg) led to a significant decrease in hepatic content of NO, and AMP by 27% and 28%, respectively, compared to the TAA group. Noteworthy, the hepatic contents of these parameters were comparable to the normal group. Moreover, compared to the control group, TAA-induced significant decline in ATP, ADP, GSH, and CoQ10 by 30%, 69%, 159%, and 34%. Conversely, the administration of CLEE (100 mg/kg) restored the hepatic content of ATP, ADP, and CoQ10 by 36%, 106%, and 38%, respectively, compared to the TAA group.

**Figure 7:**
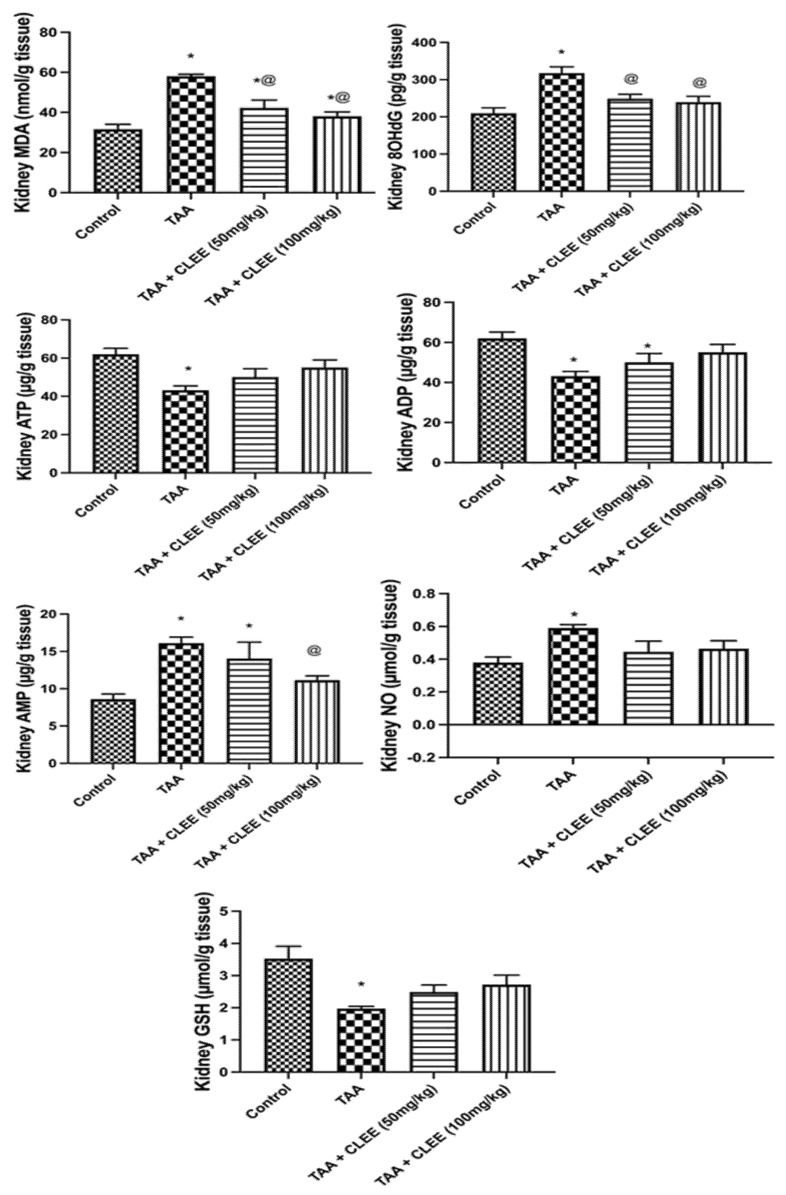
Effect of *C. laevigatus* EtOH extract (CLEE) on renal oxidative stress and energy parameters of rats intoxicated by TAA. Each value represents the mean of six animals ± SE. Statistical analysis was performed using one-way ANOVA followed by the Tukey-Kramer multiple comparisons test. (* vs. control group, and @ vs. TAA group) at *p* < 0.05.

Similarly, Figure 7 illustrated a substantial increase in renal content of MDA, 8OHdG, NO, and AMP by 83%, 52%, 88%, and 55%, respectively, owing to the effect of TAA compared to the control group. Contrarywise, the administration of CLEE (50 mg/kg) decreased the renal content of MDA and 8OHdG by 27% and 22%, respectively, compared to TAA. Meanwhile, the administration of CLEE (100 mg/kg) decreased the renal content of MDA, 8OHdG, and ATP by 34%, 25%, and 31%, respectively, compared to TAA. Additionally, compared to the control group, TAA induced a significant reduction in renal content of ATP, ADP, and GSH by 30%, 149%, and 44%. Meanwhile, the administration of CLEE (50 and 100 mg/kg) failed to reduce the renal content of ATP, ADP, and GSH compared to TAA. However, CLEE (100 mg/kg) improved these parameters to levels comparable to the normal control group.

#### 2.2.5. Histopathological Results

- Liver

**Figure 8:**
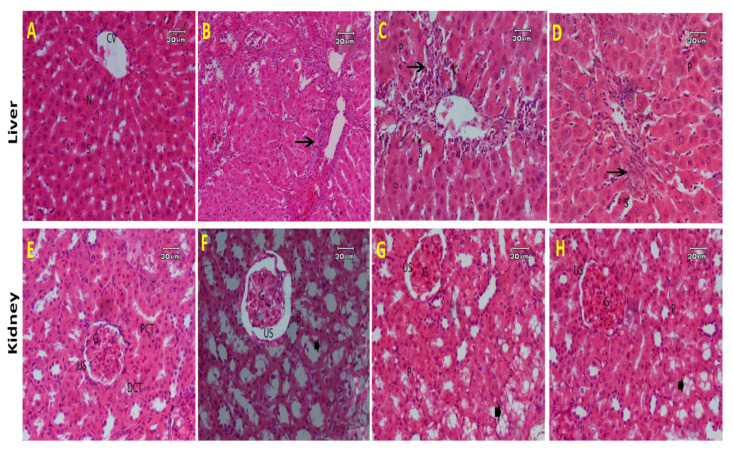
A photomicrograph of (**A**) rat liver of control group showing normal hepatic architecture. Central vein (CV), blood sinusoids (S) and nucleus (N) are found; (**B**) rat liver intoxicated by TAA showing disorganized hepatocytes, fibrosis with inflammatory cell proliferation in between the congested portal vein, aggregation of inflammatory cells (arrow), bridging of fibroblast-like cells, degeneration changes with cytoplasmic necrotic areas, and deeply pyknotic nuclei, (H & E stain, Scale bar: 60 µm); (**C**) liver from rats administered TAA and 50 mg/kg b.w. of CLEE showing mild to moderate fibrosis with thin bridging fibroblasts, inflammatory cell infiltration around central vein (arrow), degeneration, mild necrosis (arrowhead), dilated sinusoids (S), and pyknotic nuclei (P); and (**D**): liver from rats administered TAA with 100 mg /kg b.w. of CLEE showing few degenerative hepatocytes with less fibrosis and thin bridging fibroblasts, dilated sinusoids (S), and pyknotic nuclei; (**E**) kidney section of control group showing normal structure of the glomerulus (G), urinary space (US), *Proximal convoluted tubule* (PCT) and *distal convoluted tubule (DCT)*; (**F**) kidney section of rats intoxicated by TAA, showing shrunken glomeruli (**G**) with dilated capsular spaces (US), tubular epithelial cell degeneration, and necrosis associated with pyknotic nuclei (P); (**G**) kidney section of rats administered TAA and 50 mg/kg b.w. of CLEE showing moderated degeneration (arrow head), pyknotic nuclei (P) observed in tubules epithelial cells; (**H**) kidney section of rats administered TAA and 100 mg/kg b.w. of CLEE showing improved degeneration of tubules (arrow head) and pyknotic nuclei (P). (H & E stain, Scale bar: 30 µm).

The liver tissue of the normal control groups exhibited normal architecture of the central vein, blood sinusoids, and nuclei (Figure 8A). TAA-intoxicated rats showed a noticeable degree of fibrosis, hepatic damage with disruption of hepatic cell cord, and necrotic hepatocytes, with infiltration of inflammatory cells around the central veins. Moreover, inflammatory cell infiltrations in the portal areas and pseudo-lobulation were also detected. Congestion of blood vessels and degenerative changes with nuclear pyknosis were also observed (Figure 8B). The liver section of TAA and 50 mg/kg b.w. of CLEE showed mild to moderate fibrosis with necrotic hepatocytes and inflammatory cell proliferation between the congested portal veins. Degenerative changes with nuclear pyknosis and dilated blood sinusoids were also observed (Figure 8C). The liver section of TAA and 100 mg/kg b.w. of CLEE group indicated fine fibroblastic cells and few inflammatory cells infiltration surrounding and adjacent to the central vein. Degenerative changes with nuclear pyknosis and dilated blood sinusoids were also observed (Figure 8D).

- Kidney

Light microscopic pictures of renal tissues from the control group showed normal architecture of glomeruli, normal Bowman’s space in between, with normal tubular structures (*Proximal convoluted tubule* *& *distal convoluted tubule) (**Figure 8E). Histopathological examination of sections from rat kidneys intoxicated by TAA showed impaired renal morphology throughout, shrunken glomeruli with wide dilated capsular spaces, and generalized tubular epithelial cell degeneration, necrosis associated with pyknotic nuclei, and inflammatory cell infiltration (Figure 8F). Kidneys from animals administered TAA and 50 mg/kg b.w. of CLEE showed partial damage to glomeruli with less dilated Bowman’s capsule and less tubular dilation (Figure 8G). Kidneys from animals administered TAA and 100 mg/kg b.w. of CLEE showed nearly normal kidney architecture in most of the glomeruli and Bowman’s capsules space with intact epithelial cell and tubules structures in addition to mild damage of proximal and distal tubules (Figure 8H).

## 3. Discussion

In the current study, the protective effect of CLEE was investigated against hepatorenal toxicity induced by thioacetamide. TAA is a frequent hepatotoxic agent resulting in free radical production and oxidative stress through its *S*-oxide metabolite characterized by high reactivity and instability [31]. The present study illustrated the capability of CLEE to mitigate oxidative stress induced by TAA, as confirmed by a significant decrease of hepatorenal MDA, 8-OHdG, and NO contents and enhancement of GSH and CQ10 content. Moreover, CLEE reduced oxidative stress in blood as evidenced by enhancement of GSH and catalase levels and reduced levels of MDA and SOD. CLEE exhibited antioxidant activity [7] attributed to its active flavonoid components. It was reported that flavonoids had various mechanisms to combat oxidative stress, including inhibiting enzymes used in ROS formation, scavenging ROS, and regulating the antioxidant defense system [32]. Here, CLEE reduced the histopathological changes in the liver and kidney induced by TAA. Oxidative stress is closely related to the pathological damage of hepatic cells representing a typical response to chronic liver injury [33]. Liver fibrosis is a wound-healing response to hepatocellular injury. It includes deposition of an extracellular matrix by activated hepatic stellate cells (HSCs) [34].

The decrease in pathological changes could be confirmed by a decrease in MDA and/or enhancement of GSH and CoQ10 contents. MDA is a product of lipid peroxidation that affects cell membranes, causing cell injury and necrotic cell death [35]. It was concluded that GSH and CoQ10 exhibited antioxidant activity and inhibited lipid peroxidation [36,37]. There is substantial evidence that TAA-induced liver damage is linked to increased oxidative stress and tissue damage caused by free radicals. Nevertheless, CLEE reduced the pathological changes via enhancement of catalase enzyme (antioxidant enzymes). Having the ability to decompose hydrogen peroxide to water and oxygen, catalase, as a therapeutic agent, is used to manage numerous oxidative stress-related diseases [38].

The increase in serums AST, ALT, and GGT in rats administered TAA is likely due to liver injury resulting in increased plasma membrane permeability and eventual leakage of the enzymes into the blood [39]. Also, serum ALP levels were elevated due to defective hepatic excretion as a reflection of hepatobiliary and hepatocellular injury [40]. These structural changes and this oxidative damage could also affect the liver’s secretory function, resulting in hypoproteinemia and hypoalbuminemia, leading to circulatory and renal dysfunction [41]. Here, CLEE suppressed the increase in liver enzymes induced by TAA, probably via decreasing oxidative stress in blood and liver and enhancing endogenous antioxidants. These findings were in agreement with a previous study on *Cyperus laevigatus* total extract revealing that the extract at 50 mg/kg daily dose exhibited antioxidant and antidiabetic effects with no toxic symptoms or mortality in rats [7]. These results completely agreed with previous data that revealed potent protective effects of *Cyperus* plants against inflammation, hepatic damage, ulcers, and diabetes such as *C. laevigatus, C. rotundus, C. rotundus*. *C. alternifolius,* and *C. conglomeratus* [7,10].

Moreover, another proposed mechanism for CLEE protection was evidenced by resistance to increase in inflammatory markers, TNF-α, IL6, CRP, and NO levels induced by TAA, indicating anti-inflammatory properties of the extract. It has been reported that TNF-α content was increased in many conditions of cellular injury [42]. A previous experiment indicated that TNF-α directly evoked mitochondrial ROS production and DNA damage and dysfunction [43]. Nitric oxide reacts with reactive oxygen species to form reactive nitrogen species, which generates peroxynitrite, a robust biological oxidant generally implicated in toxic effects on cells and tissues. Peroxynitrite represents a mediator of protein oxidation and nitration, lipid peroxidation, mitochondrial dysfunction, and cell death [44]. The increase in IL6 levels has been implicated in liver and kidney diseases [45,46].

Elevation of liver enzymes was associated with higher CRP concentrations [47]. Moreover, CLEE alleviated the decrease in hepatorenal ATP content, suggestive of its protective effect on mitochondria against the toxic impact of TAA that inhibited the mitochondrial respiratory chain [48]. The present study showed that CLEE could protect rats against TAA-induced liver fibrosis and kidney injury probably by (i) reduction of MDA, (ii) enhancement of GSH and CoQ10; and (iii) inhibition of the increase of inflammatory markers, IL2, CPR, TNF-α, NO that evoke oxidative stress.

Secondary metabolites are the main contributors to the biological potentialities of the plant extracts [49]. Polyphenolic compounds, including flavonoids and phenolic and organic acids, were documented as the most active compounds against free radicals and thus inflammation [49,50]. Current findings reveal that flavonoids were the main components of CLEE, encompassing flavones, flavonols, aurones, and flavanonequinones.

The annotated flavonoids exhibited potent free radical scavenging effects due to the polyhydroxylation in their aromatic systems comprising mobile hydrogens [49]. Structurally, the 3′,4′-*O*- and 5- hydroxylation of the B and A rings, along with the presence of 4-carbonyl moiety in the flavonoids, were the main mediators of increasing antioxidant and anti-inflammatory potentialities [4,49,51]. These structural moieties increased the protection against oxidative stress and damage via donating hydrogen and/or electrons to radicals [4]. Through this process, they are involved in (i) stabilization of the networks of cell membranes and (ii) inhibition of the inflammatory cytokines formation and expression such as TNF-α, Transforming Growth Factor beta (TGF-*β*), and interleukins [52,53]. The present chemical profiling revealed that luteolin derivatives such as methoxylated derivatives and/or glycosides, were the main identified components having anti-inflammatory and free radicals scavenging actions [4]. Luteolin and their methoxylated derivatives and/or glycosides were reported to have principal roles in the inhibition of liver and kidney injuries and inflammation via decreasing and/or inhibition of fatty liver development [54], liver cirrhosis [55] and hepatocellular carcinoma [56], in addition to renal apoptosis, epithelial injury [57], and elevation of fructose consumption on the kidney [58].

Phenolic acids are mainly derivatives of benzoic and cinnamic acids in which the phenolic ring methyl ester represents a pharmacophore with various possibilities of interaction with different cell membrane proteins [4]. Many phenolic and organic acids were assigned in CLEE, such as ferulic, caffeic, cinnamic, and quinic acids and their derivatives. Several reports described that these phenolic acids are active metabolites for liver and renal protection via (i) freezing of the free radicals, (ii) suppressing liver cholestasis via inhibition of the matrix of extracellular gene expression [59], (iii) activation of the AMP-activated protein kinase (AMPK) or mitogen-activated protein kinase (MAPK) via enhancement of lipid metabolism [60], (iv) decreasing inflammatory cytokines formation in the kidney [61], (v) enhancing oxidative kidney status, and vi) decreasing lipid peroxidation [5].

## 4. Materials and Methods

### 4.1. Chemicals and Reagents

All the organic solvents used for extraction and chromatographic experiments, as well as the chemical reagents, were of analytical grade. The thioacetamide with a purity of 98.5%, 1,1,3,3-tetraethoxypropane (malondialdehyde), and glutathione were obtained from Sigma-Aldrich (Saint Louis, MO USA).

### 4.2. Plant Material and Extract Preparation

The whole plant of *C. laevigatus* L. was collected from Kafr Elsheikh Governorate (31°35′34.19″ N; 31°05′18.00″ E, Kafr Elsheikh) Egypt in March 2020. Ahmed M. Abdel Gawad, Professor of Plant Ecology, Botany Department, Faculty of Science, Mansoura University, Egypt, carried out the plant collection and authentication. A plant voucher specimen (Man-20-X17CL-56) was deposited in the Faculty of Science herbarium, Mansoura University, Egypt.

Afterward, 600 g of the air-dried whole plant was extracted with 70% aqueous ethanol at room temperature for five days and then filtered. This extraction step was repeated three times. All the extract was collected and then evaporated under vacuum until complete dryness afforded a dark black gum (28.3 g). The extract was kept in the refrigerator at 4 °C until the start of the analysis.

### 4.3. High-Resolution Ultra-Performance Liquid Chromatography-Mass Spectrometry Analysis (UPLC-ESI–qTOF-MS)

The UPLC-MS analysis was carried out with the same documented protocol and conditions as El-Newary, et al. [62] and Farrag, Abdallah, Khattab, Elshamy, Gendy, Mohamed, Farag, Efferth and Hegazy [6].

### 4.4. Thioacetamide-Induced Hepatorenaltoxicity Experiments

#### 4.4.1. Animals, Ethical Statement, and Acute Toxicological Studies

Adult male Wistar rats (150–160 g) were chosen from the animal house of the National Research Centre, Egypt. They were fed a standard pellet diet and water ad libitum and kept at adjusted temperature (22 ± 2 °C) with a 12 h light-dark cycle [63].

The overall study and animal handling were performed following the guidelines of the NRC ethics committee, National Institutes of Health, and Canadian Council on Animal Care [Acceptance No.: 19218]. The body weights of the rats were recorded weekly.

According to results of Elshamy, El-Shazly, Yassine, El-bana, Farrag, Nassar, Singab, Noji and Umeyama [7], CLEE was safe up to 5 g/kg b.w. without any mortality or toxic symptoms in rats.

#### 4.4.2. Grouping and Experimental Design

Twenty-four rats were left for 14 days for acclimatization and then sorted equally into four groups. **Group I** (negative control group): All the rats in this group were administered orally with an equal volume of vehicles. **Group II** (positive control group): Rats were intraperitoneal (IP) administered with 100 mg/kg b.w. of TAA dissolved in distilled water [64]. **Group III**: Rats pre-treated with intragastric CLEE (50 mg/kg b.w.) dissolved in distilled water followed by treatment with TAA (IP). **Group IV**: Rats were pre-treated with intragastric CLEE (100 mg/kg b.w.) and treated with TAA (IP). The pre-treatment of used CLEE concentrations was performed half hour before the injection of TAA. The inductions of the rats by TAA were carried out three times weekly, while they were treated with CLEE daily throughout the experiment for 8 weeks.

#### 4.4.3. Blood Samples Collection and Tissue Homogenates

At the end of the experimental period (8 weeks), the overall rats were euthanized using ethyl ether in a completely sealed container. The blood samples (3 mL each) were collected in heparinized tubes before euthanasia. The centrifugation for 15 min at 3000 rpm was carried out to isolate the plasma. The liver and kidney were rapidly removed and washed in ice-cooled saline. A weighted part of each tissue was homogenized with ice-cooled saline (0.9% NaCl) to prepare homogenate. The homogenate was centrifuged at 3000 rpm for 10 min at 5 °C using a cooling centrifuge [65]. The supernatant was used for various analyses. The remaining portion of the liver or kidney was fixed immediately in 10% neutral buffered formalin.

#### 4.4.4. Liver and Kidney Function

The estimation of the liver and kidney functions, including the AST, ALT, ALP, GGT, total protein, creatinine, and uric acid levels, were performed using the kits produced by Salucea BV Medical Company (Haansberg, Netherlands).

#### 4.4.5. Plasma Oxidative Stress and Inflammatory Markers

The MDA, GSH, SOD, and catalase were evaluated calorimetrically using the kits produced by Elabscience Biotechnology Inc. (Houston, Texas, USA). The levels of MDA, SOD, reduced GSH, and total protein were assessed and measured according to the described methods and techniques of Ohkawa, et al. [66], Beutler, et al. [67], Nishikimi, et al. [68] and Lowry, et al. [69], respectively. TNF-α, IL6, and CRP were assayed by enzyme-immunoassay using a kit manufactured in R&D Systems, McKinley Place NE, Minneapolis, USA.

#### 4.4.6. Hepatic and Renal Oxidative Stress and Energy Parameters

The estimation of the MDA, 8OHdG, NO, GSH, ATP, ADP, AMP, and CoQ10 parameters were performed using the HPLC system of Agilent HP 1200 series (Santa Clara, CA, USA) that consisted of a quaternary pump, a column oven, Rheodine injector, 20 μL loop, and UV variable wavelength detector. The resulting chromatogram identified the sample concentration compared to the standard purchased from Sigma Aldrich (Missouri, MO, USA).

#### 4.4.7. Histopathologic Investigation

The liver and kidneys were dissected directly after the rats’ sacrifice for histopathological investigations. Aliquots of the liver and kidney tissues were fixed in 10% formalin in phosphate-buffered normal saline for 7 days. Then they were washed for 2 h under running tap water and dehydrated gradually in ethanol, followed by embedding in the paraffin wax. After that, the sections were de-paraffinized using xylene and stained with hematoxylin and eosin (H&E, 4–5 µm thick) (Mc, 1946). A light microscope (Olympus CX41, San Marcos, TX, USA) and SC100-digital camera attached to the computer system were used for the examination.

### 4.5. Statistical Analysis

The variability of results was expressed as means ± standard error of means (n = 3, SE). Data were evaluated by one-way analysis of variance (ANOVA) followed by Tukey-Kramer multiple comparisons. The level of significance was accepted at *p* < 0.05. All the statistical assays were carried out using Graphpad Prism-8 (San Diego, CA, USA).

## 5. Conclusions

UPLC-ESI-QTOF–MS/MS analysis of CLEE derived from the whole plant resulted in the identification of 94 compounds belonging to various classes with relatively high concentrations of flavonoids and phenolic acid derivatives. Luteolin and tricin derivatives represented the main constituents in *Cyperus laevigatus*. CLEE exhibited antioxidant and anti-inflammatory effects against TAA-induced toxicity owing to the synergistic action of major phytochemicals detected in the former. A high dose of CLEE at 100 mg/kg b.w exhibited a higher protective effect against hepatorenal damage than did its lower dose at 50 mg/kg b.w. The possible mechanism of CLEE on the liver and kidney might implicate regulation of energy metabolism, oxidative stress, and inflammatory markers. The present findings reveal the protective effects of the main constituents of *Cyperus laevigatus* against hepatic and renal injuries. The results also suggest further and deeper biological evaluation to enrich the scientific background regarding using CLEE to manage various diseases. However, phytochemical studies should be evaluated to isolate and identify the significant compounds in CLEE, thus determining their precise mode of action and safety.

## Figures and Tables

**Table 2:** Effect of *C. laevigatus* ethanol extract (CLEE) on body weight of rats administered with TAA.

	Weeks	Initial	1st	2nd	3rd	4th	5th	6th
Groups								
Control		145.33 ± 2.512	182.33 ± 4.958	220.67 ± 5.226	252.33 ± 3.621	273.67 ± 5.038	287.83 ± 3.728	275.5 ± 7.535
TAA		151.67 ± 3.073	173.17 ± 4.483 *	178.33 ± 4.295 *	203 ± 6.088 *	195.83 ± 4.915 *	209.83 ± 3.42 *	201.67 ± 4.072 *
TAA + CLEE (50 mg/kg)		157 ± 2.251	195.83 ± 3.516	211.83 ± 11.923	223.67 ± 9.821 *	221.17 ± 10.199 *	227.17 ± 14.885 *	240 ± 10.724 *
TAA + CLEE (100 mg/kg)		150.83 ± 3.745	187 ± 11.204	195.83 ± 11.677	207.33 ± 11.67	213.33 ± 11.029 *	224.17 ± 9.411 *	224.83 ± 5.036 *@

Each value represents the mean of six animals ± SE. Statistical analysis was performed using one-way ANOVA followed by the Tukey-Kramer multiple comparisons test. (* vs. control group, and @ vs. TAA group) at *p* < 0.05.

## Data Availability

Not applicable.

## Sample Availability

Samples of the compounds are not available from the authors.
